# Acquisition of respiratory surface EMG: a systematic literature review of electrode configurations and methodological reporting

**DOI:** 10.1186/s13054-025-05696-x

**Published:** 2025-11-07

**Authors:** R. S. P. Warnaar, J. E. Francovich, L. van Baaren, D. W. Donker, A. H. Jonkman, E. Oppersma

**Affiliations:** 1 Cardiovascular and Respiratory Physiology, Technical Medical Centre, University of Twente, Technohal 3184, P.O. Box 217, 7500 AE Enschede, The Netherlands; 2 Department of Intensive Care Medicine, Erasmus University Medical Centre, Rotterdam, The Netherlands; 3 Intensive Care Department, University Medical Center Utrecht, Utrecht, The Netherlands

**Keywords:** Respiratory failure, Respiratory surface electromyography, Electrode configuration

## Abstract

**Background:**

Mechanical ventilation provides life-saving support to patients with respiratory failure, but inadequately tailored settings can lead to respiratory muscle dysfunction and poor patient outcomes. Surface electromyography (sEMG) offers a non-invasive modality to monitor respiratory muscle function. However, variability in acquisition setups limits the comparability of study findings and hinders broad clinical implementation. Therefore, we systematically appraised setup rationales and reporting quality in respiratory sEMG literature.

**Methods:**

The MEDLINE ALL, Embase, and Web of Science databases were systematically searched on 19 September 2024 for studies reporting original respiratory sEMG data in adults during spontaneous breathing. sEMG methodology was extracted in accordance with the reporting guidelines of the International Society of Electrophysiology and Kinesiology and analyzed by target muscle and medical domain.

**Results:**

240 out of 402 unique articles were included. The diaphragm was the most studied respiratory muscle (61%) with 48 unique setups out of 160 descriptions. Diaphragm setups with small inter-electrode distances (IEDs) were most common (n = 138, 86%). Large IED setups were predominantly applied in ICU (n = 8, 36%) and COPD (n = 5, 23%) populations. Setups for non-diaphragmatic respiratory muscles typically featured one or two dominant positions grounded in methodological studies. Reporting quality was low with a median of 5 out of 10 recommended items documented.

**Conclusion:**

This review reveals substantial diversity of diaphragm sEMG setups, reflecting differences in clinical contexts and study populations. The setups for extra-diaphragmatic muscles were more consistent and methodologically grounded. Muscle- and context-specific guidelines are essential to improve consistency and support clinical implementation of respiratory sEMG.

**Supplementary Information:**

The online version contains supplementary material available at 10.1186/s13054-025-05696-x.

## Introduction

Mechanical ventilation (MV) provides life-saving support for patients with respiratory failure. The primary goal of MV is to ensure sufficient ventilation and oxygenation, while unloading the respiratory muscles. However, MV can also cause respiratory muscle dysfunction which may, in part, reflect a mismatch of ventilatory support and patient needs. In this sense, mechanisms contributing to the development of respiratory muscle dysfunction include ventilator over- and ventilator underassistance, with patient-ventilator asynchrony (PVA) also a potential adverse factor [1]. In the intensive care unit (ICU), diaphragm dysfunction is associated with prolonged weaning from MV, prolonged ICU stays, and increased mortality [2–9]. In the context of chronic respiratory failure, home mechanical ventilation (HMV) is frequently complicated by PVA, leading to reduced comfort, more dyspnea, and impaired sleep quality [10, 11]. As a result, there is growing recognition of the importance to monitor respiratory muscle activity to better guide and tailor ventilatory support [12].

Surface electromyography (sEMG) offers a promising non-invasive approach to respiratory muscle monitoring, allowing for large-scale monitoring ranging from quantifying respiratory effort in ICU patients to detecting PVAs in HMV settings [13]. Respiratory sEMG is acquired by applying electrodes to the skin, facilitating ease of use in both acute and chronic care settings. However, clinical implementation has long been limited by technical challenges, particularly signal contamination from cardiac activity and crosstalk from adjacent muscles [14].

Recent advances in signal processing, including automated quality assessment [15], have improved the interpretability of respiratory sEMG and paved the way for a broader clinical use. Yet, the comparability of sEMG recordings depends heavily on consistent acquisition setups [16]. For non-respiratory muscles, standardization efforts such as the Surface ElectroMyoGraphy for the Non-Invasive Assessment of Muscles (SENIAM) initiative [17] and the ‘Consensus for experimental design in electromyography’ (CEDE) project [18] have been established. However, such standardization efforts are lacking for respiratory muscles [19, 20]. We set out to systematically appraise the current literature on respiratory sEMG acquisition setups, with a focus on the rationales behind the setups, quality of reporting, and differences across clinical domains.

## Methods

This review was designed in accordance with the Preferred Reporting Items for Systematic reviews and Meta-Analysis (PRISMA) guideline with a checklist available in Additional file 1.

### Study identification and selection

A comprehensive search was performed using MeSH terms and free text variations of the core concepts ‘sEMG’ of ‘respiratory muscles’ in ‘adult’ subjects. The MEDLINE ALL, Embase, and Web of Science literature databases were systematically searched from database creation until 19 September 2024. The full search queries can be found in Additional file 2. Original research articles were eligible for inclusion if they reported the use of original sEMG data to study respiratory muscles, i.e., inspiratory or expiratory, in adults during spontaneous breathing. Articles were excluded if the full-text was not available, if it was not written in English, or if it studied only singing, swallowing, non-breathing related muscle activation, or (neuromuscular) stimulation induced muscle activity. The reference lists of eligible studies were searched for additional articles meeting the screening criteria.

Title and abstract screening, as well as full-text review were performed in Covidence (Covidence systematic review software, Veritas Health Innovation, Melbourne, Australia). Title and abstracts were screened independently by two reviewers (LvB, JF), full-texts by one reviewer (LvB). Disagreements were resolved by discussion.

### Data extraction

sEMG methodology was extracted from the included articles in Covidence using a predefined online extraction form (Additional file 3). Data on the sEMG setup included the positions of the active and reference electrodes, inter-electrode distance (IED), skin preparation, and electrode properties (manufacturer, shape, size, material), as based on the reporting guidelines of the International Society of Electrophysiology and Kinesiology (ISEK) for sEMG studies [21]. The rationale for the selected measurement setup was also noted. Study population characteristics were documented regarding sex, patient condition, and type of ventilatory support. Outcomes that were not explicitly documented were classified as ‘Not Reported’. Data was initially extracted by a single reviewer (LvB). A second reviewer (RW) independently extracted data from a random subset comprising 10% of the included studies for cross-validation. Conflicts were resolved by discussion and by cross-referencing against the original article.

### Data analysis

Electrode positions and electrode-pair orientations, i.e., anode-to-cathode orientation, were tabulated per muscle and aggregated in pivot tables. Combinations of electrode position and electrode-pair orientation were analyzed per application and medical domain, and for trends over time. Quality of methodological reporting was assessed in accordance with ISEK guidelines [19, 21, 22], evaluating the proportion of studies reporting on the individual items and the number of sEMG setup items reported per study. Each item was scored on an all-or-nothing basis, requiring that the item be provided for all reported muscles. If an item was missing for any muscle, it was marked as ‘Not Reported’.

## Results

### Study identification

**Fig. 1:**
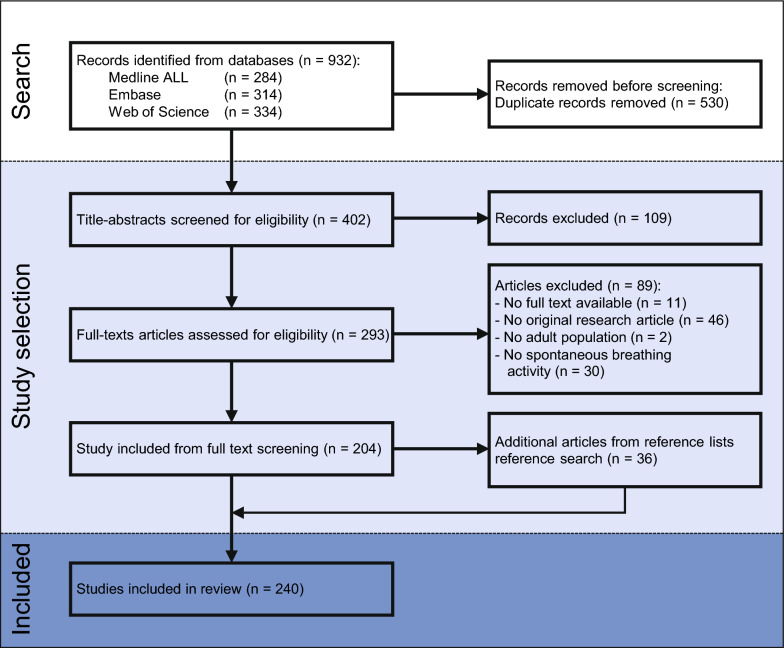
Study selection flow diagram

A total of 402 unique publications were identified, of which 109 and 89 publications were excluded during the title-and-abstract and full-text screening, respectively, yielding 204 eligible articles (Fig. 1). An additional 36 articles were identified by reference screening, resulting in a total of 240 included articles. The main reasons for exclusion during the full-text screening were the absence of original sEMG data (n = 46) and not studying spontaneous breathing activity (n = 30).

**Fig. 2:**
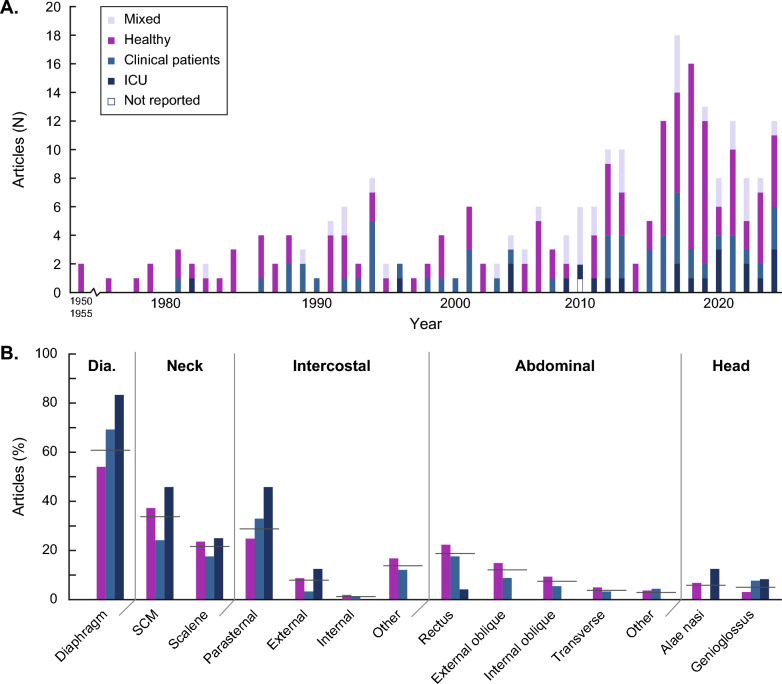
Publication counts, study populations, and studied muscles. **A** Annual publication counts by population type. **B** Proportion of articles per population type reporting respiratory sEMG for the specified muscles. Horizontal lines indicate the proportion over all included articles. Abbreviations: ICU: Intensive Care Unit; Di: Diaphragm; SCM: Sternocleidomastoid

The increasing publication counts over time show that respiratory sEMG is emerging in research, with half of the articles published in the last decade (Fig. 2). The majority of studies were conducted on healthy subjects only (n = 127, 53%). The 240 included articles studied a grand total of 5454 patients with the majority being male (3327, 66%). The use of sEMG in clinical studies outside the ICU, parallelled the development of sEMG in healthy subjects, rising from the 1980 s, and formed 23% (n = 54) of all included studies. Respiratory sEMG studies in the ICU started rising in 2005 and made up 9% (n = 22) of included articles. An additional 14% (n = 34) conducted respiratory sEMG research in mixed populations comprising both healthy and clinical participants. Clinical populations included subgroups of chronic obstructive pulmonary disease (COPD, n = 31, 36%), sleep medicine and sleep apnea (n = 11, 13%), neuro-muscular disorder (NMD, n = 16, 18%), and asthma (n = 5, 6%). The remaining 25 clinical studies (28%) covered other pulmonological, cardiological and surgical patients.

### Electrode positions

The diaphragm was the most studied respiratory muscle (n = 146, 61%), followed by the sternocleidomastoid (SCM, n = 81, 34%), the parasternal (n = 69, 29%), and the scalene (n = 52, 22%) muscles (Fig. 2b). 55 studies (23%) measured sEMG of at least one abdominal muscle. The largest number of electrode positions was found for the diaphragm with 50 unique reproducible positions out of 146 studies. Seven unique reproducible positions were identified across 81 studies for the SCM, four out of 52 for the scalene, and eight out of 69 for the parasternal muscles. The extracted electrode positions for all respiratory muscles are listed in Additional File 4.

**Fig. 3:**
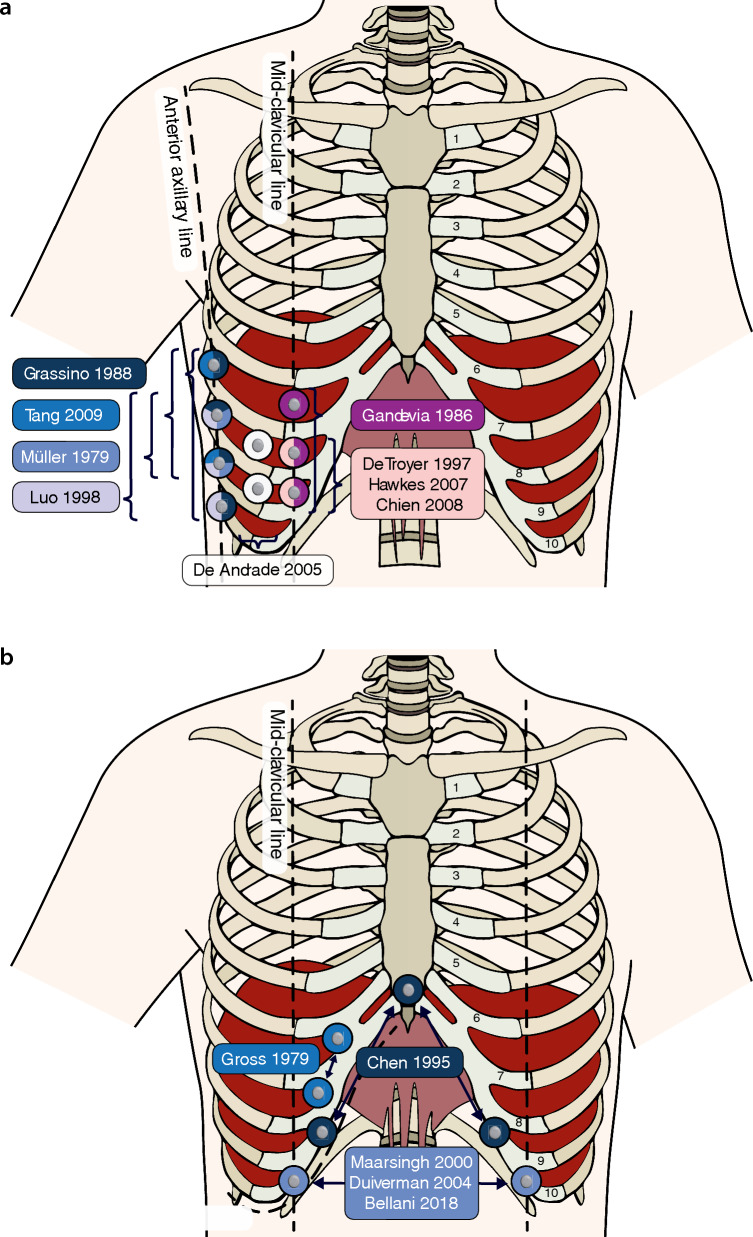
Diaphragm electrode positions. **A** Electrode positions at longitudinal anatomical lines. **B** Electrode positions on or near the costal margin. Costal numbers are indicated on the costal cartilages

For the diaphragm, setups were categorized as small or large IED based on the spatial distribution of the electrodes relative to their anatomical landmarks. In small IED setups, electrodes were placed on ribs, over the intercostal spaces (ICS) or on the costal margin at a single longitudinal anatomical line (Fig. 3A, Additional File 5), typically the anterior-axillary line (AAL, n = 32), the mid-clavicular line (MCL, n = 17), or in between the MCL and AAL (n = 10). In large IED setups (Fig. 3B), electrodes were typically placed bilaterally at the crossing of the costal margin with the MCL (n = 9) or nipple line (n = 4), or at the xiphoid and 15 to 16 cm from the xiphoid along the costal margin (n = 4). Out of 22 articles using a large IED setup, 13 studies were conducted in the ICU (n = 8, 36%) and in COPD patients (n = 5, 23%). Studies of the diaphragm in other clinical domains were more scarce (NMD: 10, sleep medicine: 8, asthma: 3), and demonstrated a mixed pattern of small and large IED setups (Table 1, Additional files 4 and 5).

**Table 1:** Reference article groups and citations counts for diaphragm electrode positions

Diaphragm electrode position	Study type	Healthy	Clinical populations					ICU	Total^†^
			**COPD**	**Asthma**	**OSA**	**NMD**	**Other**		
Gross et al. [23]: 6th-7th ICS at CM (Ri)	Diaphragmatic fatigue in healthy subjects	13 (50%)	6 (23%)				9 (35%)	1 (4%)	26
Maarsingh et al. [24] and Duiverman et al. [25]: Nipple line bilaterally at CM	Diaphragm activity in healthy, COPD, and (pediatric) asthma patients	8 (38%)	5 (24%)			2 (10%)	3 (14%)	7 (33%)	21
Luo et al. [26]: 7th/8th/9th ICS at AAL (Ri and Le)	Compound muscle action potentials under phrenic nerve stimulation in healthy subjects	14 (88%)		2 (13%)	2 (13%)	3 (19%)	3 (19%)		16
Grassino [27]: 6th/7th/8th/9th ICS at AAL (Ri)	Book on respiratory muscles in COPD	7 (88%)	1 (13%)						8
Chen et al. [28]: Xiphoid-5 cm to 16 cm at CM, bilaterally	Phrenic nerve conduction (amplitude and latency) in healthy subjects	7 (100%)		2 (29%)			1 (14%)		7
De Troyer [29]: 7th/8th ICS close to CM at MCL (Ri)	Invasive diaphragm EMG (discharge rate) in healthy and COPD patients	4 (57%)			1 (14%)	1 (14%)	1 (14%)	2 (29%)	7
Tang* [30]: 6th-8th ICS at AAL (Ri)	Respiratory drive and effort in OSA	1 (20%)	3 (60%)				2 (40%)		5
De Andrade [31]: 7th/8th ICS at MCL-AAL (Ri)	Inspiratory muscle training in healthy elderly and stable COPD patients	4 (100%)							4
Hawkes [32]: Lowest ICS at MCL (Ri)	Diaphragm activity during acute non-fatiguing, submaximal inspiratory muscle loading in healthy subjects	1 (33%)		1 (33%)		2 (67%)			4
Chien [33]: 7th/8th ICS at MCL (Ri)	Reliability of diaphragm and external intercostal sEMG in response to cervical magnetic stimulation	4 (100%)			1 (25%)	1 (25%)	1 (25%)		4
Bellani et al. [34]: MCL bilaterally at CM	Diaphragm activity in ICU patients						1 (33%)	2 (67%)	3
Gandevia [35]: 6th/7th ICS to CM 5 cm below at MCL (Ri)	Effect of lung volume on compound muscle action potentials under phrenic nerve stimulation in healthy subjects					2 (67%)	1 (33%)	2 (67%)	3
Müller** [36]: 7th-8th ICS at AAL (Ri)	Diaphragm muscle tone in sleep	3 (100%)	1 (33%)						3

Percentages reflect the proportion of citations from each subpopulation relative to the total number of citations for the corresponding reference article

*Source article in Chinese, electrode positions derived from citing articles ** Also Nipple line bilaterally at CM, but not cited as such

^†^Counts for population types add up to more than the total citation counts because of studies with mixed population types

Abbreviations: CM Costal margin; ICS intercostal space; MCL Mid clavicular line; AAL anterior axillary line; (Ri) right sided; (Le) left sided; ICU intensive care unit; COPD chronic obstructive pulmonary disease; OSA obstructive sleep apnea; NMD: neuro-muscular disorder. X^th^/Y^th^/Z^th^ ICS: X^th^, Y^th^, or Z^th^ intercostal space; X^th^—Y^th^: X^th^ to Y^th^ intercostal space

The electrode positions for the SCM varied in the studied muscle segment, where the middle third was most commonly studied (n = 29), followed by the upper third (n = 14) and lower third (n = 7). Other variations were the upper half (n = 2), the lower half (n = 1), the full muscle (n = 1), and the junction of the sternal and clavicular heads to 2–5 cm cranially (n = 4). The scalene electrode positions demonstrated subtle variations with all positions located in the posterior triangle of the neck. Main locations were at the level of the cricoid to overlie the anterior scalene (n = 18), behind the clavicular fibres of the SCM just above the clavicle to overlie the medial scalene (n = 7).

For the parasternal muscles, 38 out of 69 studies (55%) used unilateral electrode placement, of which 19 cases placed both electrodes in the second intercostal space. Placement of the most medial electrode varied from directly next to the sternum to a distance of 3 cm from the sternal edge. Other common positions were in the second and third ICS on the parasternal line (n = 8), in the second ICS and on the nearby sternum or an adjacent rib (n = 5), and in the first and second ICS on the parasternal line (n = 3). 16 studies reported a bilateral placement, with electrodes in the second intercostal space both on the left and on right side at 3 cm from the sternal edge.

### Rationale for sEMG setups

The used sEMG setup was substantiated by 189 (78.8%) studies, most commonly referencing a previous article (n = 169). Twelve electrode positions were cited more than twice for the diaphragm when traced back to the original reference (Table 1), with Gross et al. [23], Maarsingh et al. and Duiverman et al. [24, 25], and Luo et al. [26] cited more than ten times. Specific sEMG setup use, indicated by these citation counts, varied among clinical contexts and study populations. Gross et al. is highly cited for measurements in healthy subjects (n = 13, 50%) and a heterogeneous set of other clinical patients (n = 9, 35%). The bilateral setup described by Maarsingh et al. and Duiverman et al. was more commonly referenced by studies in COPD (n = 5, 24%) and ICU (n = 7, 33%) patients, whereas the setup by Luo et al. was referenced most by studies in healthy subjects (n = 14, 88%). A maximum of three reference papers were cited more than twice for non-diaphragmatic respiratory muscles (Table 2).

**Table 2:** Reference article groups and citation counts for non-diaphragmatic electrode positions

Muscle	Electrode position	Study type	N
Parasternal intercostal	Maarsingh et al. [24] & Duiverman et al. [25]: 3 cm from sternum bilaterally	Parasternal activity in healthy, COPD, and (pediatric) asthma patients	16
Sternocleidomastoid	Falla et al. [37]: Sternal head: Lower third parallel to sternal-notch mastoid line Clavicular head: Direction of head behind sternal head	Innervation zones of the sternocleidomastoid to provide guidelines for electrode positioning	10
Scalene	Duiverman et al. [25]: Bipolar electrodes left and right on the neck over the scalene	Scalene activity in healthy, COPD, and (pediatric) asthma patients	13
	Falla et al. [37]: Lower third parallel to clavicular head of sternum	Innervation zones of the scalene to provide guidelines for electrode positioning	10
Abdominal muscles	Maarsingh et al. [24] & Duiverman et al. [25]: Rectus abdominis: 2 pairs lateral of umbilicus, 4 cm apart	Diaphragm activity in healthy, and COPD patients. Abdominal muscle sEMG for detecting crosstalk in diaphragm lead	7
	McGill et al. [38]: Rectus abdominis: 3 cm lateral of umbilicus External oblique: 15 cm lateral of umbilicus in transverse direction Internal oblique: Below External Oblique, just above inguinal ligament	Compare intramuscular to surface EMG in healthy subjects for deep muscles in the abdominal wall	6
	Ng [39]: Rectus abdominis: - Upper (above umbilicus): 2° inferolateral to the midline - Lower (below umbilicus): 8° inferomedial to the midline External oblique: - Most inferior point of the CM on the line to the contralateral pubic tubercle (with underlying internal oblique) - Parallel to eight ICS near the costal cartilage (without underlying internal oblique) Internal oblique: triangle bounded by the inguinal ligament, lateral border of rectus sheath and a line joining both anterior superior iliac spines	Cadaver study on muscle fiber orientation of abdominal muscles for sEMG electrode positioning	5
Genioglossus	Jeffries [40]: On either side of the midline 1 cm apart and halfway between the chin and hyoid bone	Relation between respiratory muscle EMG and blood gas abnormality severity in pediatric OSA patients	5
Alae nasi	Strohl et al. [41]: On either side of the lateral wall of the external nares	Timing of alae nasi activity relative to flow onset during wakefulness, quiet sleep, and CO_2_-induced hyperpnea	4
	Connel [42]: Bilaterally on the anterolateral aspect of the nose	Effects of nasal airflow and resistance on alae nasi activity during exercise in healthy subjects	3

A total of 57 studies provided a rationale beyond referencing previous articles. Anatomical or physiological rationales were provided by 23 studies, considering overlapping or adjacent muscles, muscle fiber orientation and the muscle’s zone of innervation. The right hemithorax was preferred in nine studies to minimize cardiac interference. Other studies confirmed electrode positions using ultrasound (n = 9), either to identify the muscle belly location or the area with the least amount of overlying tissue. Two studies measured on both sides and selected the most reliable signal for analysis.

### Signal acquisition reporting

**Fig. 4:**
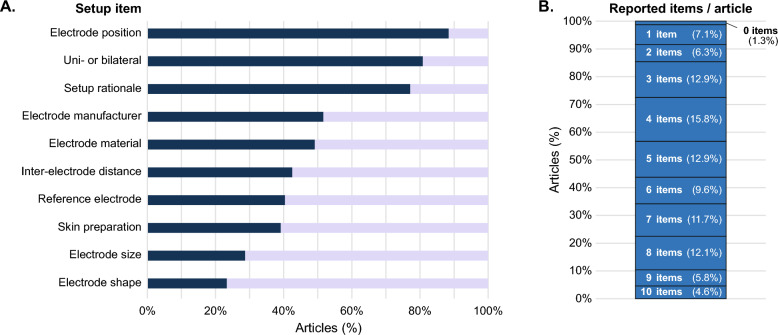
Signal acquisition reporting quality. **A** Proportion of articles reporting individual signal acquisition items. **B** Distribution of the total number of reported signal acquisition items

Only 11 studies reported all checklist items with a median of 5 reported items per study (Fig. 4). Shape (n = 56, 23%) and size (n = 69, 29%) of the electrodes were the least reported items. Although electrode positions were reported most frequently (n = 212, 88%), the descriptions commonly lacked sufficient detail to ensure reproducibility. For instance, 61 out of 160 (38%) electrode positions were not reproducible for the diaphragm. The electrode-pair orientation was described insufficiently in 54 cases, whereas the anatomical position, i.e., the combination of anatomical landmarks resulting in one unique position, was missing in 30 cases. Of the studies reporting electrode material (n = 118, 49%), Ag/AgCl electrodes were used in 92% (n = 109). Other electrode types were used infrequently (gold-plated: 6, stainless steel: 2, platinum: 1) and were primarily applied in specialized contexts. The reporting quality remained low after the year 2000, i.e., when the SENIAM guidelines were established [17], with 34.9% of studies reporting less than 5 items. The reported items per study can be found in Additional file 4.

## Discussion

This review systematically evaluated respiratory muscle sEMG acquisition setups, with a focus on electrode positioning, the rationale behind setup choices, and the quality of methodological reporting. Diaphragm electrode positions demonstrated substantial diversity, whereas the setups for other respiratory muscles were more consistent, typically featuring one or two dominant positions per muscle. Overall, sEMG setups were insufficiently reported, with ambiguous descriptions of electrode positions and frequent underreporting of skin preparation, IED, and electrode characteristics. The development of guidelines specifically for respiratory electromyography could promote standardization, enabling comparable findings across studies and ultimately widespread adoption of respiratory sEMG in research and daily clinical practice.

### Tailoring electrode positions to clinical contexts

Respiratory sEMG offers a non-invasive method to enable monitoring of respiratory muscle activity in a larger number of patients, to optimally inform tailoring of ventilatory support from ICU to HMV settings [13]. The clinical utility of sEMG largely depends on the acquisition of high-quality signals, which is critically influenced by electrode placement. Achieving this requires balancing two goals: capturing a representative signal from the target muscle and minimizing interference from surrounding muscles [43]. This balance is particularly challenging for the diaphragm due to its complex anatomy. The diaphragm’s dome-shaped structure lies close to the body surface, near its attachment to the ribs and sternum, offering a broad range of potential electrode positions. However, not all positions are equally suitable across clinical contexts, depending on the parameters of interest, the clinical setting, and physiological conditions. These factors likely contributed to the diversity of observed diaphragm electrode positions.

Respiratory sEMG has been predominantly applied in studies on healthy subjects or clinically stable patients. In these controlled settings, the small IED setups of Gross and Luo were cited frequently [23, 26], building on their original applications in fundamental neurophysiology. These setups were initially used to assess diaphragm fatigue [23] and evaluate compound muscle action potentials following phrenic nerve stimulation [26]. Both applications require the analysis of subtle temporal and spectral features, such as the median power frequency, and latency, that are highly sensitive to electrode spacing, electrode-pair orientation, and proximity to the muscle [16, 43, 44]. Small IEDs are not merely suitable in these cases, but essential to ensure accurate signal capture. The broader adoption of these small IED setups may consequently reflect their compliance with SENIAM and CEDE guidelines [16, 17], which supports their use even beyond their originally intended applications.

While small IED setups can yield high-quality signals in ideal conditions, with consistent breathing patterns and minimal movement artifacts, their performance is easily compromised in more challenging environments. This is particularly true in settings like the ICU or in patients with respiratory failure, where altered breathing patterns, patient agitation, and interference from clinical equipment are common. In these contexts, signal quality, particularly the signal-to-noise ratio (SNR), is often compromised by motion artifacts and abdominal muscle crosstalk [15, 45]. Large IED setups offer practical advantages in such conditions, as they enhance the sEMG amplitude and SNR, making them more resilient to motion artifacts and abdominal muscle crosstalk [43, 46]. Additionally, electrode placement near the costal margin may reduce electrode movement relative to the diaphragm, as this region is most likely to overlie the diaphragm’s zone of apposition – even when lung volume increases up to total lung capacity [47, 48]. As a result, diaphragm muscle fibers in this region maintain the most consistent distance and inclination relative to the skin, allowing for optimally stable sEMG recordings. These advantages likely contributed to the more widespread adoption of large IED setups in ICU and COPD research, as reflected by the frequent use and citation of the setups described by Maarsingh and Duiverman [24, 25], Chen [28], and Bellani [34]. These differences in electrode configuration across clinical contexts illustrate how electrode preferences are shaped by both physiological and practical demands. Rather than enforcing a one-size-fits-all approach, standardization of diaphragm sEMG acquisition should be guided by domain-specific considerations reflecting the unique challenges and goals of each application. The initial aim of such standards is to reduce heterogeneity within domains, improving comparability of studies with a specific application and supporting the integration of respiratory sEMG into routine clinical practice.

The development of such guidelines requires clear recognition of the methodological limitations and strengths associated with different electrode configurations. Small IED setups, with their narrow pickup volume, offer superior spatial resolution and isolation of the target muscle activity [43]. This allows for detection of subtle changes in muscle activity as required for fatigue assessment or phrenic nerve stimulation studies [23, 26]. The limited spatial coverage of small IED setups moreover reduces the likelihood of capturing crosstalk, yet any contamination has a proportionally larger impact due to the lower signal amplitudes [43]. In contrast, large IED setups expand the pickup volume, which may be advantageous for deeper or less accessible muscles, such as the diaphragm in obese patients. While this also increases the likelihood of capturing crosstalk, the higher signal amplitudes often reduce its relative impact [43, 46]. Larger IEDs additionally attenuate higher-frequency components due to spatial filtering [49], which is especially important in respiratory muscles given the significant spectral overlap between EMG and cardiac activity in the lower frequency ranges [50, 51]. Furthermore, electrode pair orientation and placement near the motor end-plate can further reduce EMG accuracy [17, 43], especially in large IED setups. These trade-offs highlight that no single configuration is universally optimal. Instead, setup selection should reflect a deliberate balance between signal specificity and robustness, tailored to the physiological and practical demands of the clinical context.

In contrast to the diaphragm, electrode placement for other respiratory muscles tends to be more consistent. The most frequently cited studies for these muscles were specifically designed to investigate the sEMG feasibility or optimize electrode configurations, i.e., for the SCM [37], scalene [37], parasternal [25] and abdominal muscles [38, 39]. These methodological studies provide a solid foundation and could serve as reference standards for future research within the described contexts. For smaller muscles like the genioglossus and alae nasi, the reference studies were primarily physiological in nature [40–42]. Despite their different focus, these studies provide clear and consistent electrode placement descriptions that could support standardization efforts for these muscle groups.

### Signal acquisition reporting

Despite the availability of CEDE and SENIAM guidelines, comprehensive reporting of respiratory sEMG signal acquisition remains uncommon [17, 18]. While electrode positions are generally reported to some extent, reproducibility is limited due to missing or ambiguous critical details, e.g., the exact anatomical landmarks and electrode-pair orientation. Terminological inconsistencies further complicate interpretation, with terms like ‘reference’, ‘ground’, and ‘common’ used interchangeably, at times even contradicting standard definitions [52]. These issues are further complicated by the limited reporting of other critical acquisition parameters, including skin preparation, IED, and electrode characteristics (size, shape, material). Although often perceived as minor, these factors significantly influence signal quality. For example, large electrode diameters and large IEDs act as spatial low-pass filters, attenuating high-frequency components of the signal [53, 54]. Similarly, electrode material and skin preparation influence impedance and SNR [55].

Inadequate reporting not only hampers reproducibility but also slows clinical translation, as observed in other fields such as exercise and sports physiology, where sEMG adoption has been limited by similar issues [56]. To advance respiratory sEMG toward clinical application, future studies should adhere to established guidelines and provide complete, unambiguous description of acquisition details. Ideally, these should be supported by schematic illustrations to clearly convey electrode configurations. In addition, example tracings and quality measures such as baseline level and SNR are proposed to be added to demonstrate overall signal quality.

### Towards clinical standardization

Methodological heterogeneity and underreporting are known challenges observed in sEMG literature, both for respiratory muscles, as well as in other domains like pediatrics, neurophysiology, and exercise and sports physiology [19, 20, 56–59]. Notably, the field of clinical gait analysis is making significant strides towards standardizing sEMG acquisition, likely facilitated by the collaborative efforts of national and international societies [60]. Similar initiatives in respiratory sEMG could be fostered by organizations like the European Respiratory Society (ERS) and the American Thoracic Society (ATS), whose current statements acknowledge the clinical utility of respiratory sEMG but do not yet provide methodological standards [61, 62].

### Strengths and limitations

This review addresses multiple clinical populations and muscle groups, enabling a cross-domain synthesis of setup patterns and reporting practices. This broader scope allowed us to identify consistent setups for extra-diaphragmatic muscles and substantial heterogeneity for diaphragm recordings. Previous systematic reviews have noted similar reporting deficiencies, but were typically confined to specific domains, such as healthy subjects [59], conscious individuals [19], or ICU patients [20], limiting their ability to compare trends across clinical domains.

The search was limited to the MEDLINE ALL, Embase, and Web of Science, which offer broad coverage of biomedical and clinical literature. While databases like PEDro and SPORTDiscus were not searched, potentially limiting representation from rehabilitation and sports science, the selected sources align with this review’s focus on methodological variation across clinical domains. The search strategy was defined to specifically include non-invasive recordings of EMG, rather than applying explicit exclusions. Some relevant studies may have fallen out of the search scope by not explicitly reporting ‘surface EMG’ in searchable fields. This is particularly relevant for extra-diaphragmatic muscles, where surface EMG is typically the standard approach and its non-invasive nature is often left unspecified, e.g., for the parasternal muscles [63, 64]. Yet, the synthesis of setup patterns remains robust, as electrode positions for these muscles were found to be more consistent across studies.

To ensure data accuracy, a cross-validation of 10% of included studies (25 studies, 1039 extracted items) was performed, revealing four major and three minor extraction conflicts. While the full dataset was not cross-validated, the low frequency and limited scope of these inconsistencies support the reliability of the extraction protocol.

The majority of included articles were conducted in healthy subjects, whereas the clinical clusters were smaller in number despite spanning a wide range of clinical populations. The limited number of studies within subgroups may limit the generalizability of dominant electrode setups for those specific populations, particularly when reflecting the preferred practices of individual research groups rather than broader consensus. Additionally, most studies included predominantly male participants, which may further restrict generalizability to female or mixed-sex populations.

This review appraised reported sEMG setups to facilitate standardization but did not assess signal quality, as quantitative metrics are rarely included in published studies. Comparative analysis would have relied on visual interpretation of exemplary sEMG tracings, introducing interpretive bias. Additionally, the formation of coherent analytical clusters for such analysis would have been complicated by methodological variability and underreporting. Electrode positions were grouped primarily by anatomical region rather than by electrode-pair orientation, leading to within-group variation that likely affects signal quality. Rather than relying on speculative comparisons, findings were interpreted in the context of studies that directly investigated the impact of electrode configuration on signal quality [43, 46], providing a more robust foundation for understanding setup diversity and guiding future standardization.

### Future directions

Future studies are needed to compare acquisition setups within specific clinical domains to inform methodological standardization in respiratory sEMG. These studies should include clinically relevant, representative cohorts, accounting for a potential overrepresentation of male subjects identified in this review. Beyond evaluating signal characteristics, such as amplitude and SNR, it is crucial to assess how well the signal reflects the physiological process under study, e.g., breathing effort or fatigue, to ensure clinical relevance alongside technical performance.

## Conclusion

This review highlights substantial variability in respiratory sEMG acquisition setups, particularly for the diaphragm, where electrode configurations appear to reflect differences in clinical context and study populations. Small IED setups were commonly used in controlled environments involving healthy subjects, while large IED setups were more prevalent in ICU and COPD studies. In contrast, electrode positions for other respiratory muscles were more consistent. Methodological reporting frequently lacked adherence to CEDE standards, with critical details highly underreported or described inconsistently, such as anatomical landmarks, electrode-pair orientation, interelectrode distance, and electrode characteristics. This limits respiratory sEMG reproducibility and hinders cross-study comparisons. The development of domain-specific standards for respiratory sEMG acquisition is essential to advance the field. Such guidelines would provide an explicit, citable reference to guide acquisition choices, reduce methodological diversity, and ultimately support the integration of respiratory sEMG into routine clinical care.

## Supplementary Information

Additional file1

Additional file2

Additional file3

Additional file4

Additional file5

## Data Availability

The datasets used and/or analysed during the current study are available upon reasonable request.

## Abbreviations

MV: Mechanical Ventilation
PVA: Patient-Ventilator Asynchrony
ICU: Intensive Care Unit
HMV: Home Mechanical Ventilation
sEMG: Surface Electromyography
SENIAM: Surface ElectroMyoGraphy for the Non-Invasive Assessment of Muscles
CEDE: Consensus for Experimental Design In Electromyography
PRISMA: Preferred Reporting Items for Systematic reviews and Meta-Analysis
ISEK: International Society of Electrophysiology and Kinesiology
COPD: Chronic Obstructive Pulmonary Disease
NMD: Neuro-Muscular Disorder
SCM: Sternocleidomastoid
IED: Inter-Electrode Distance
ICS: Intercostal Space
AAL: Anterior Axillary Line
MAL: Mid-Axillary Line
MCL: Mid-Clavicular Line
ECL: External Clavicular Line
CM: Costal Margin
Ri: Right sided
OSA: Obstructive Sleep Apnoea
SNR: Signal-to-Noise Ratio
PEEP: Positive End-Expiratory Pressure
ERS: European Respiratory Society
ATS: American Thoracic Society
