# Supplementary material for: Acquisition of respiratory surface EMG: a systematic literature review of electrode configurations and methodological reporting
Source: Crit Care. 2025 Nov 7;29:476. doi: 10.1186/s13054-025-05696-x (PMC12595820; doi:10.1186/s13054-025-05696-x)
Supplement: Supplementary file 2 — Additional file2 [file 13054_2025_5696_MOESM2_ESM.docx]

# Additional file 2 - Search strategy

## Search query

| DB | Query | #Hits |
| --- | --- | --- |
| MEDLINE ALL | ((Electromyography/ AND Skin/) OR (sEMG OR ((transcut* OR surface) ADJ3 (electromyogra* OR EMG*))).ab,ti,kf.)  AND (breathing muscle/ OR (((breath* OR airway* OR expirat* OR inspirat* OR intercostal OR respirat*) ADJ3 (muscle*)) OR diaphragm).ab,ti,kf.)  NOT ((exp child/ OR exp infant/ OR pediatrics/ OR adolescent/) NOT exp adult/)  NOT (congres* OR abstract*).pt.  AND english.la. | 284 |
| Web of Science | TS=(  ((sEMG OR ((transcut* OR surface) NEAR/2 (electromyogra* OR EMG*))))  AND ((((breath* OR airway* OR expirat* OR inspirat* OR intercostal OR respirat*) NEAR/2 (muscle*)) OR diaphragm))  NOT ((juvenil* OR adolescen* OR preadolescen* OR youth* OR child* OR schoolchild* OR minors OR teen OR teens OR teenager* OR infan* OR toddler* OR pediatr* OR paediatr* OR puber* OR baby OR babies OR girl* OR boy* OR newborn* OR neonat* OR premature* OR pre-matur* OR kid OR kids OR underag* OR kindergar* OR pubescen* OR prepubesc* OR school* OR preschool* OR highschool* OR suckling OR PICU OR NICU OR PICUs OR NICUs) NOT (adult* OR elderl* OR man OR men OR woman OR women OR frail* OR octagener* OR geriat* OR ((old*) NEAR/3 (patient* OR peopl*))))  )  NOT DT=(Meeting Abstract OR Meeting Summary)  AND LA=(English) | 334 |
| Embase | ('surface electromyography'/de OR (electromyography/de AND 'skin electrode'/de) OR (sEMG OR ((transcut* OR surface) NEAR/3 (electromyogra* OR EMG*))):ab,ti,kw)  AND ('breathing muscle'/exp OR (((breath* OR airway* OR expirat* OR inspirat* OR intercostal OR respirat*) NEAR/3 (muscle*)) OR diaphragm):ab,ti,kw)  NOT ('juvenile'/exp NOT 'adult'/exp)  NOT ([Conference Abstract]/lim OR [Conference Review]/lim)  AND [ENGLISH]/lim | 314 |
