# Supplementary material for: Acquisition of respiratory surface EMG: a systematic literature review of electrode configurations and methodological reporting
Source: Crit Care. 2025 Nov 7;29:476. doi: 10.1186/s13054-025-05696-x (PMC12595820; doi:10.1186/s13054-025-05696-x)
Supplement: Supplementary file 3 — Additional file3 [file 13054_2025_5696_MOESM3_ESM.pdf]

Preview

Full text data

Patients (number, (male, female))

Applies to all fields : If the data cannot be found in the article/is not reported write: "NR"

Patients (type)

Clinical in this context refers to: 'not Healthy'. These can both be ambulatory or hospitalized patients/subjects.

Use the OTHER option to specify the specific disease or patient group

1. ☐ Healthy
2. ☐ Clinical patients
3. ☐ ICU
4. ☐ Other

Ventilation

Use the OTHER option to specify the exact type of ventilation used

1. ☐ None
2. ☐ Non-invasive
3. ☐ Invasive
4. ☐ Other

Electrode/sensor placement

Muscle groups

1. ☐ Parasternal intercostal
2. ☐ Internal intercostal
3. ☐ External intercostal
4. ☐ Intercostal (not otherwise specified)
5. ☐ Diaphragm
6. ☐ Scalene
7. ☐ Sternocleidomastoid
8. ☐ Alae nasi
9. ☐ Genioglossus
10. ☐ External obliques

11. ☐ Internal obliques
12. ☐ Obliques (not otherwise specified)
13. ☐ Transverse abdominis
14. ☐ Rectus abdominis
15. ☐ Not reported
16. ☐ Other

Skin preparation

Electrode location

If multiple muscles are analyzed specify the muscle which matches the description

Uni- or bilateral

This is often unclear. Articles often refer to bilateral placement when they're measuring unilaterally but on both sides.

For Example: unilateral measurement for parasternal muscles could be: "Electrode 1: 2nd ICS right side, electrode 2: 3rd ICS right side"

Doing this measurement on both hemithoraxes would be still be unilateral. You can specify "left", "right" or "both sides".

Bilateral measurement would be: "Electrode 1: 2nd ICS right side, electrode 2: 2nd ICS left side"

If this distinction cannot be made with enough certainty write: "NR"

Reference electrode

Motivation (for placement location)

Often a reference, sometimes one for each specific muscle. Most often found in "Methods" section adjacent to description of electrode position. Sometimes other motivations for electrode positions are (also) mentioned in the discussion.

Electrode/sensor properties

Manufacturer

Shape

Size

Material

Inter-electrode distance

COMMENTS

Blank space for comments or interesting findings/specifications in articles that are not already reported in the previous fields.
