# Supplementary material for: Acquisition of respiratory surface EMG: a systematic literature review of electrode configurations and methodological reporting
Source: Crit Care. 2025 Nov 7;29:476. doi: 10.1186/s13054-025-05696-x (PMC12595820; doi:10.1186/s13054-025-05696-x)
Supplement: Supplementary file 5 — Additional file5 [file 13054_2025_5696_MOESM5_ESM.docx]

# Additional file 5 – Supplementary tables and figures

Supplementary Table 1 Diaphragm positions with small inter-electrode distances

|  | | **Height** | | | | | | | | | | | | | |
| --- | --- | --- | --- | --- | --- | --- | --- | --- | --- | --- | --- | --- | --- | --- | --- |
|  |  | **ICS** | | | **ICS to ICS** | | | | | | |  |  |  |  |
|  |  | **7^th^/8^th^** | | **Other** | **6^th^-7^th^** | | **7^th^-8^th^** | | **6^th^-8^th^** | | **Other** | **Xiphoid** | **Other**** | **Unknown** | **Total** |
| **Width** | **AAL** | 2 | 4 | | 5 | 8 | | 7 | | 1 | |  | 5 |  | 32 |
|  | **MAL** |  | 2 | | 1 | 2 | |  | | 4 | |  | 0 |  | 9 |
|  | **MCL** | 6 | 5 | | 2 |  | |  | | 1 | |  | 3 |  | 17 |
|  | **MCL/AAL** | 6 |  | |  | 4 | |  | |  | |  |  |  | 10 |
|  | **Costal margin** |  |  | | 16 |  | |  | |  | |  |  |  | 16 |
|  | **Other*** | 1 | 5 | | 3 | 9 | |  | |  | | 7 | 3 | 2 | 30 |
|  | **Unknown** |  | 3 | | 3 |  | |  | |  | |  | 4 | 14 | 24 |
|  | **Total** | 15 | 19 | | 30 | 23 | | 7 | | 6 | | 7 | 15 | 16 | 138 |

* Other widths include: Parasternal line, Nipple line, Mid-axillary to anterior axillary line (MAL/AAL), Mid-axillary to mid-clavicular line (MAL/MCL), Mid-axillary to external clavicular line (MAL/ECL), Xiphoid

** Other heights include: ICS: 5^th^, 6^th^, 7^th^, 8^th^, 9^th^, lowest, and 6^th^ or 7^th^; ICS to ICS: 5^th^ to 6^th^, 8^th^ to 9^th^, 10^th^ to 11^th^, 5^th^ to 7^th^, and 7^th^ to 9^th^; and Others: 7^th^ ICS to costal margin, 7^th^ to 4 cm below, FRC and TLC diaphragm level, 5 à 10 cm below the xiphoid, and 12^th^ rib margin.

Abbreviations: AAL: Anterior axillary line; MAL: Mid-axillary line, MCL: Mid-clavicular line; ECL: Extra-clavicular line; MCL/AAL In between MCL and AAL; ICS: Intercostal space

Supplementary Figure 1 – Number of articles per population type reporting respiratory sEMG for the specified muscles. Abbreviations: ICU: Intensive Care Unit; COPD: Chronic obstructive pulmonary disease; SCM: Sternocleidomastoid; NMD: Neuro-muscular disorder
